# The Protective Effect of Magnesium Lithospermate B on Hepatic Ischemia/Reperfusion *via* Inhibiting the Jak2/Stat3 Signaling Pathway

**DOI:** 10.3389/fphar.2019.00620

**Published:** 2019-05-31

**Authors:** Ning Zhang, Li Han, Yaru Xue, Qiangqiang Deng, Zhitao Wu, Huige Peng, Yiting Zhang, Lijiang Xuan, Guoyu Pan, Qiang Fu

**Affiliations:** ^1^Department of Pharmacology of Chinese Materia Medica, China Pharmaceutical University, Nanjing, China; ^2^Shanghai Institute of Materia Medica, Chinese Academy of Sciences, Shanghai, China; ^3^University of Chinese Academy of Sciences, Beijing, China

**Keywords:** magnesium lithospermate B, hepatic ischemia/reperfusion, acute injury, anti-inflammation, Jak2/Stat3 signaling pathway

## Abstract

Acute inflammation is an important component of the pathogenesis of hepatic ischemia/reperfusion injury (HIRI). Magnesium lithospermate B (MLB) has strong neuroprotective and cardioprotective effects. The purpose of this study was to determine whether MLB had underlying protective effects against hepatic I/R injury and to reveal the potential mechanisms related to the hepatoprotective effects. In this study, we first examined the protective effect of MLB on HIRI in mice that underwent 1 h ischemia followed by 6 h reperfusion. MLB pretreatment alleviated the abnormal liver function and hepatocyte damage induced by I/R injury. We found that serum inflammatory cytokines, including IL-6, IL-1β, and TNF-α, were significantly decreased by MLB during hepatic ischemia/reperfusion (I/R) injury, suggesting that MLB may alleviate hepatic I/R injury *via* inhibiting inflammatory signaling pathways. Second, we investigated the protein level of p-Jak2/Jak2 and p-Stat3/Stat3 using Western blotting and found that MLB could significantly inhibit the activation of the Jak2/Stat3 signaling pathway, which was further verified by AG490 in a mouse model. Finally, the effect of MLB on the Jak2/Stat3 pathway was further assessed in an *in vitro* model of RAW 264.7 cells; 1 µg/ml LPS induced the secretion of inflammatory mediators, including IL-6, TNF-α, and activation of the Jak2/Stat3 signaling pathway. MLB significantly inhibited the abnormal secretion of inflammatory factors and the activation of the Jak2/Stat3 signaling pathway in RAW264.7 cells. In conclusion, MLB was found for the first time to reduce inflammation induced by hepatic I/R *via* suppressing the Jak2/Stat3 pathway.

## Introduction

Hepatic ischemia/reperfusion injury (HIRI) is a major complication during various diseases and liver surgery procedures, such as hemorrhagic shock, trauma, liver transplantation, and hepatectomy (Arkadopoulos et al., 2011; Douzinas et al., 2012; Lu et al., 2018). HIRI is characterized by high morbidity and mortality, which is attributed to the fact that oxidative stress, immune responses, and cell apoptosis are activated by ischemia/reperfusion (I/R) (Bzeizi et al., 1997; Van Golen et al., 2012; Van Golen et al., 2013). Currently, ischemic preconditioning and pharmacological agents are employed in clinics to prevent and mitigate I/R injury (Li et al., 2015). However, ischemic preconditioning is effective only in some young patients (Clavien et al., 2003), and the efficacy of available drugs is limited. There is no well-established method to prevent and mitigate I/R injury, which will improve the safety of major liver surgery and liver transplantation (Robertson et al., 2017). Thus, there is an urgent need to find effective methods/drugs for the treatment of hepatic I/R injury.

The dominant pathogenesis for HIRI involves two phases: the early process of ischemia-induced hepatocyte injury and the successive process of reperfusion-induced immune response (Ju et al., 2016; Zhang et al., 2018). During the ischemic process, damage-associated molecular patterns (DAMPs), such as reactive oxygen species (ROS), DNA fragments, nuclear factors, cytosolic proteins, and others, are released from the dead cells (Lu et al., 2018; Mihm, 2018). DAMPs will be recognized by Toll-like receptors (TLRs) on the cell membrane and then activate hepatic resident macrophages, Kupffer cells (KCs) (Chang and Toledo-Pereyra, 2012). KCs will secrete excessive proinflammatory cytokines to cause high levels of hepatocyte death, exacerbate liver damage, and even lead to systemic inflammatory response syndrome and multiple-organ failure (Guo, 2013; Triantafyllou et al., 2018). Suppression of the inflammatory response may be a powerful way to reduce I/R-induced hepatic injury.

According to previous studies, in addition to TLR recognition of DAMPS, the Janus kinase/signal transducers and activators of the transcription (Jak/Stat) signaling pathway plays a vital role in the inflammatory responses (Wang et al., 2015a; Roskoski, 2016; Rahimifard et al., 2017; Bousoik and Montazeri Aliabadi, 2018); Jak2 and Stat3 are the most important family members of the Jak and Stat proteins. Mutations in the Jak and Stat genes lead to multiple immune syndromes (Banerjee et al., 2017). The expression of cytokines is impacted due to the change in the Jak/Stat signaling pathway (Gurzov et al., 2016). In addition, the Jak2/Stat3 signaling pathway participates in the multiple-organ damage caused by I/R, such as brain (Hu et al., 2017), myocardial (Chen et al., 2019), and renal I/R injury (Luo et al., 2016). Organ damage was alleviated by changing Jak2/Stat3 activation or phosphorylation.

**Figure 1:**
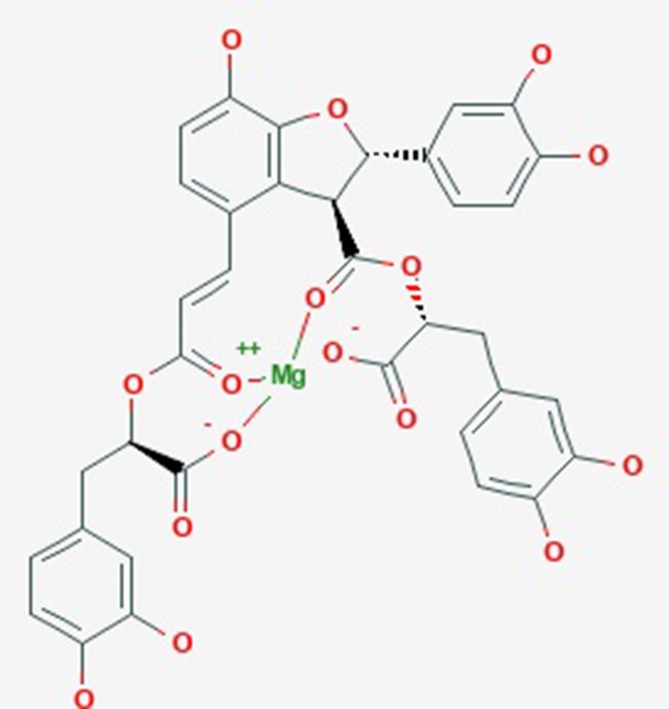
The molecular structure of magnesium lithospermate B obtained from PubChem substance SID: 135075733.

Magnesium lithospermate B (MLB, **Figure 1**) is a water-soluble component and is extracted from the traditional Chinese medicine *Salvia miltiorrhiza Bunge*, known as DanShen, which has been used to cure cardiac–cerebral vascular disease and chronic renal failure (Zhou et al., 2005; Bu et al., 2013; Huang et al., 2018). In past studies, substantial scientific evidence has suggested that MLB could protect against stroke (Cao et al., 2015), myocardial infarction (Du et al., 2010), and depression (Quan et al., 2015). Meanwhile, MLB could protect against neuroinflammation induced by lipopolysaccharide (LPS) in BV2 microglial cells and inhibit the inflammatory response *via* inhibiting the nuclear factor-kappa B signaling pathway in activation T cells (Cheng et al., 2012; Tai et al., 2018). It is unclear whether the anti-neuroinflammatory efficacy of MLB could help alleviate hepatic I/R damage.

In this study, we established HIRI in mice to investigate whether MLB could ameliorate this condition. The potential mechanisms of MLB anti-I/R in the liver were investigated, especially from inflammatory response perspectives.

## Materials and Methods

### *In Vivo* Experiment

#### Animals

The animals used in our study were obtained from the Shanghai Laboratory Animal Co. (Shanghai, China). Male C57BL/6 mice weighing 22–24 g and aged 6–8 weeks were housed in a specific pathogen-free environment with air-conditioned animal quarters at a controlled temperature of 23 ± 1.5°C and a relative humidity of 70 ± 20%. The mice were fed *ad libitum* with laboratory chow.

All animal experiments were approved by the Institutional Animal Care and Use Committee of Shanghai Institute of Materia Medica, Chinese Academy of Sciences.

### Animal Surgery

All animals underwent sham operations or hepatic I/R surgery. A warm partial (∼70%) hepatic I/R model was conducted as previously described (Castellaneta et al., 2014). In brief, mice were anesthetized by injection intraperitoneally (i.p.)with pentobarbital sodium (50 mg/kg). The animals were laparotomized, and the portal vein, hepatic artery, and bile duct were clamped with an atraumatic vascular clip blocking blood supply to the median and left lateral lobes of the liver. The sham mice were only laparotomized without hepatic ischemia. After 60 min of hepatic ischemia, the clip was removed, and the blood supply was restored. After 6 h of reperfusion, blood was drawn from the hearts of mice under isoflurane anesthesia, and liver tissues were collected.

### Drug Treatment

MLB (purity ≥ 99%) was kindly provided by Professor Lijiang Xuan (Shanghai Institute of Materia Medica, Chinese Academy of Sciences). It was administered by the intravenous route (30 mg/kg body weight, dissolved in sterile physiological saline solution) 24 h, 12 h, and 1 h before surgery. The Jak2 inhibitor AG490 (12 mg/kg body weight) was obtained from Selleck Chemical (Houston, TX, USA) and dissolved in 5% DMSO and 95% PBS. AG490 was administered i.p. as a positive control.

### Blood and Tissue Samples

All blood samples were centrifuged (3,000 rpm, 4°C) for 15 min to obtain serum stored at −80°C for biochemistry analyses. The liver tissues were collected, and parts were stored at −80°C for Western blot analysis, while others were immediately fixed in 10% formalin for hematoxylin-and-eosin staining.

### Blood Biochemical Analyses

Alanine aminotransferase (ALT), aspartate aminotransferase (AST), and lactate dehydrogenase (LDH) levels in serum were measured by a standard clinical automatic analyzer (SYSMEX JCA-BM6010C) in the laboratory of the Chinese National Compound Library.

### Hematoxylin–Eosin Staining

Three or four liver tissues were randomly selected for pathology analysis. Briefly, the fixed liver tissues were embedded in paraffin wax, and then, 4-μm-thick liver sections were cut for the next experiment. The prepared sections were stained with hematoxylin and eosin (H&E). The morphology results were assessed by a pathologist who was blinded to the experimental groups. The liver injury score was determined according to Suzuki’s method (Ge et al., 2017).

### Detection of Superoxide Dismutase Activity and Malondialdehyde Production

Appropriate liver tissues were lysed using the specific lysate, and the supernatants were separated from the homogenization by centrifugation (12,000×*g*, 10 min, 4°C) to detect superoxide dismutase (SOD) activity and MDA content. SOD activity and malondialdehyde (MDA) content were detected using the SOD and MDA assay kit (Beyotime Biotechnology, Jiangsu, China) according to the manufacturer’s instructions.

### *In Vitro* Experiment

#### Cell Culture

RAW264.7 macrophage cells were kindly provided by Professor Likun Gong, Shanghai Institute of Materia Medica, Chinese Academy of Sciences. Cells were cultured with DMEM containing 10% (v/v) FBS at 37°C in an atmosphere of 5% CO_2_.

### Cell Viability Assays

Cells were seeded into 96-well plates (3 × 10^5^ cells/ml) for 12 h prior to the experiment. After incubation with different concentrations of MLB (0.1–500 μg/ml) for 24 h, Cell Counting Kit-8 (CCK-8, Yeasen Biotech Co. Ltd., Shanghai, China) was used to determine the cell viability according to the manufacturer’s instructions. Briefly, 10 μL CCK-8 was added to each well and incubated for 1–4 h at 37°C, and then, the absorbance of each well was measured at 450 nm using a microplate reader (BioTek, USA).

### Measurement of Nitric Oxide in Culture Medium

Cells were seeded into six-well plates (3 × 10^5^ cells/ml) for 12 h before the experiment. After incubation with or without AG490 (75 μM) or different concentrations of MLB for 2 h, RAW264.7 cells were stimulated with 1 µg/ml LPS (Sigma Aldrich, USA) for 8 h. The nitric oxide (NO) content was investigated by Griess reagent according to the manufacturer’s instructions on the NO assay kit (Beyotime Biotechnology, Jiangsu, China). In brief, cell media were mixed with an equal volume of Griess reagent, and the absorbance of the mixture was measured using a microplate reader (BioTek, USA) at 540 nm. All experimental results were repeated at least five times independently. The concentrations of nitrite can be relatively calculated to use the content of NO in cell media.

### Enzyme Linked Immunosorbent Assays (ELISAs)

ELISA kits, obtained from R&D and Multi Science, were used to detect the concentrations of proinflammatory factors, including interleukin-6 (IL-6), interleukin-1β (IL-1β), and tumor necrosis factor-α (TNF-α), in the serum and culture medium, respectively. All the studies were conducted according to the specific manufacturer’s instructions. The absorbance was tested using a microplate reader (BioTek, USA) at 450 nm. All data are shown as pictograms per milliliter serum (pg/ml).

### Western Blot Assay

For protein extraction, the frozen liver tissues or cell samples were lysed using cold RIPA lysis buffer (Beyotime Biotechnology, Jiangsu, China) with 1% protease inhibitor cocktail (Bimake, Shanghai, China), and the supernatants of liver tissue homogenates or cell lysis solutions were obtained by centrifugation at 12,000 rpm for 10 min. The protein concentrations were quantified using a BCA assay kit (Thermo, USA) according to the manufacturer’s instructions. Equal amounts of protein samples were intermixed with sodium dodecyl sulfate (SDS)–loading buffer (Yeasen Biotech Co. Ltd, Shanghai, China) and then boiled for 10 min at 100°C. Subsequently, protein samples were separated by 10% SDS–polyacrylamide gel electrophoresis and then transferred onto polyvinylidene difluoride (PVDF) membranes (Millipore, USA) with Trans-Blot system (Bio-Rad, Hercules, CA). The membrane was blocked for 1 h with 3% bovine serum albumin (BSA) in Tris-buffered saline (TBS) containing 0.1% Tween 20 (TBST) at room temperature and then incubated overnight at 4°C with the primary antibodies. The membranes, washed with TBST three times, were treated with horseradish peroxidase (HRP)–labeled secondary antibody for 1 h at room temperature. Finally, the bands were identified using chemiluminescence (ECL) kits (Yeasen Biotech Co. Ltd, Shanghai, China). The signal intensity of the target bands was determined with Image-Pro Plus (IPP).

### Statistical Analysis

GraphPad Prism 5.0 software was used for statistical analyses, and all data are expressed as mean ± SD. The results were obtained by one-way analysis of variance (ANOVA) followed by Tukey’s multiple-comparison tests. There was a statistically significant difference when the P value was less than 0.05.

## Results

### Effect of MLB on Liver Damage Induced by I/R

**Figure 2:**
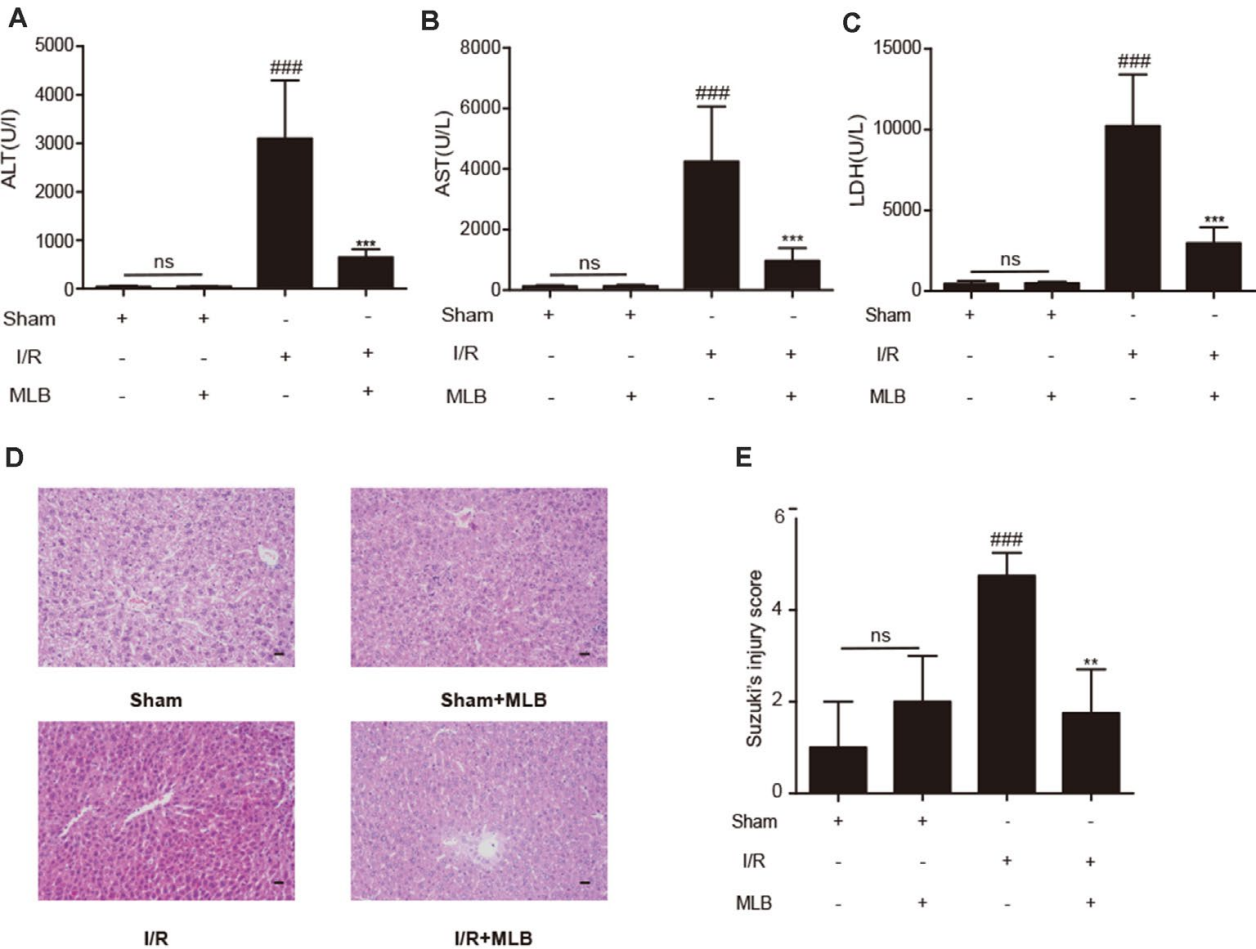
MLB pretreatment attenuates liver injury caused by ischemia–reperfusion. **(A–C)** The levels of ALT, AST, and LDH in sham, I/R, and MLB pretreatment groups. **(D**, **E)** Classical images of sham, I/R, and MLB treatment groups were examined by H&E staining, and histopathological score was calculated by Suzuki’s scoring standard, magnification x200, scale bar 50μm. Data shown are mean ± SD; ns: no statistical significance; ^###^P < 0.001 versus sham group; **P < 0.01, and ***P < 0.001 versus I/R group; n = 5 for each group.

ALT, AST, and LDH in mouse serum were examined using a standard clinical automatic analyzer. The ALT, AST, and LDH levels (**Figure 2A–C**) were not significantly different in the sham+MLB group compared to the sham group, suggesting that MLB has no adverse effect on the function of the mouse liver. The animals in the I/R group had significantly elevated ALT, AST, and LDH contents in the serum. MLB pretreatment significantly reduced the serum ALT, AST, and LDH levels in the mice after I/R surgery. Liver pathological changes were mainly revealed as varying degrees of hepatocellular swelling/necrosis, sinusoidal/vascular congestion, and inflammatory cell infiltration. The changes were more severe in the I/R group than in the sham group (**Figure 2D**) and were reversed in the MLB pretreatment group. The pathological score of the MLB group was significantly lower than that of the I/R-alone group (**Figure 2E**). These results suggest that MLB could protect against I/R-induced hepatic injury.

### Effect of MLB on Oxidative Stress in Liver and Proinflammatory Mediators in Serum

**Figure 3:**
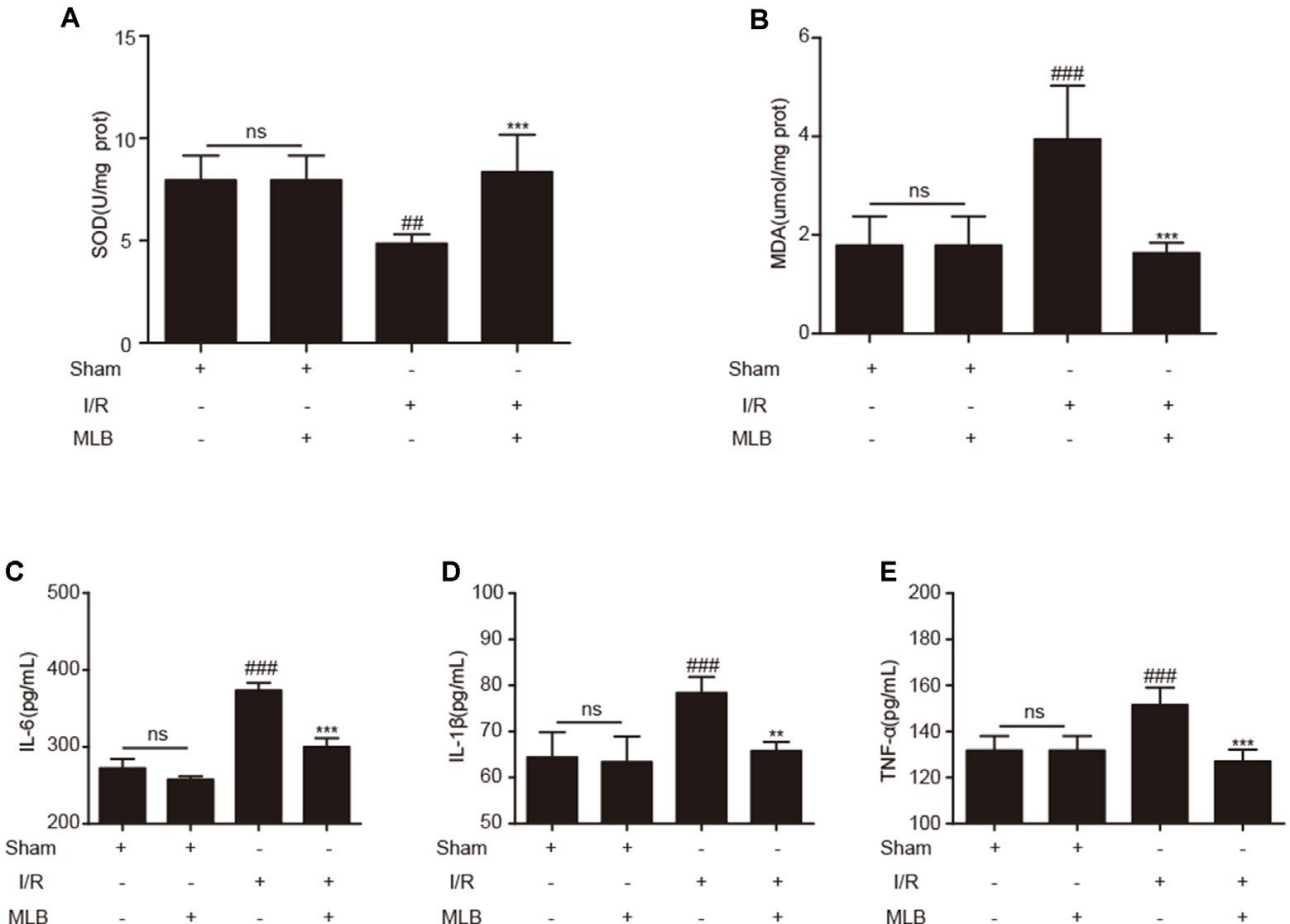
MLB pretreatment affected oxidative stress in liver and inhibited inflammatory factors’ expression. **(A**, **B)** The levels of SOD and MDA in liver tissue of sham, I/R, and MLB pretreatment groups. **(C**–**E)** Serum IL-6, IL-1β, and TNF-α were detected by ELISA in sham, I/R, and MLB group. The data are expressed as mean ± SD; ns: no statistical significance; ^##^P < 0.01, and ^###^P < 0.001 versus sham group; **P < 0.01, and ***P < 0.001 versus I/R group; n = 5 for each group.

To evaluate the effects of MLB on oxidative stress, the concentrations of SOD and MDA in liver tissue were measured. SOD and MDA concentrations did not change significantly in the sham+MLB group (**Figure 3A, B**). The concentrations of SOD and MDA were significantly changed due to I/R. MLB pretreatment could alleviate these changes and reduce oxidative stress. Serum IL-6, IL-1β, and TNF-α in the sham+MLB group were not significantly altered compared with the sham group (**Figure 3C–E**). However, serum IL-6, IL-1β, and TNF-α concentrations were abnormally elevated in the I/R group, and MLB pretreatment significantly decreased the concentrations of serum IL-6, IL-1β, and TNF-α throughout the observation period.

### Effect of MLB on Jak2/Stat3 Signaling Pathway

**Figure 4:**
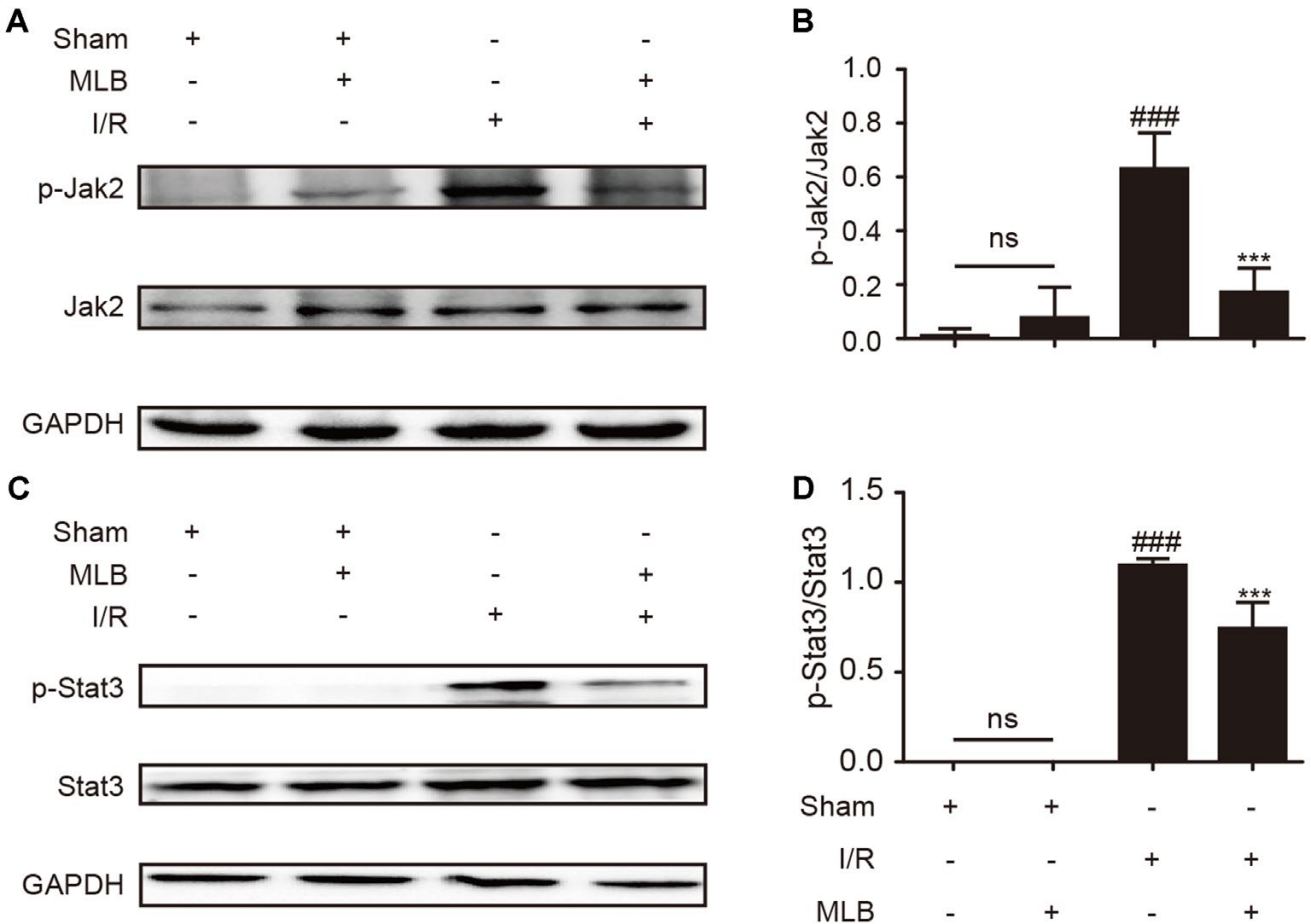
MLB pretreatment inhibited Jak2/Stat3 signal pathway in the liver tissue of I/R. **(A)** Western blot was utilized to detect the level of p-Jak2. **(B)** The quantification of p-Jak2/Jak2 was statistically analyzed. **(C)** Western blot was utilized to detect the level of p-Stat3. **(D)** The quantification of p-Stat3/Stat3 was statistically analyzed. The data are expressed as mean ± SD; n = 5 for each group; ns: no statistical significance; ^###^P < 0.001 versus sham group; ***P < 0.001 versus I/R group.

To explore the underlying mechanism of MLB on HIRI, we detected the ratio of p-Jak2/Jak2 and p-Stat3/Stat3 in the liver tissue of I/R animals. As described in **Figure 4A–D**, p-Jak2 and p-Stat3 levels were significantly higher in the I/R group versus the sham group. After MLB pretreatment, there was a significant decrease in the levels of p-Jak2 and p-Stat3.

### Effect of AG490 or MLB on Hepatic Injury Induced by I/R

**Figure 5:**
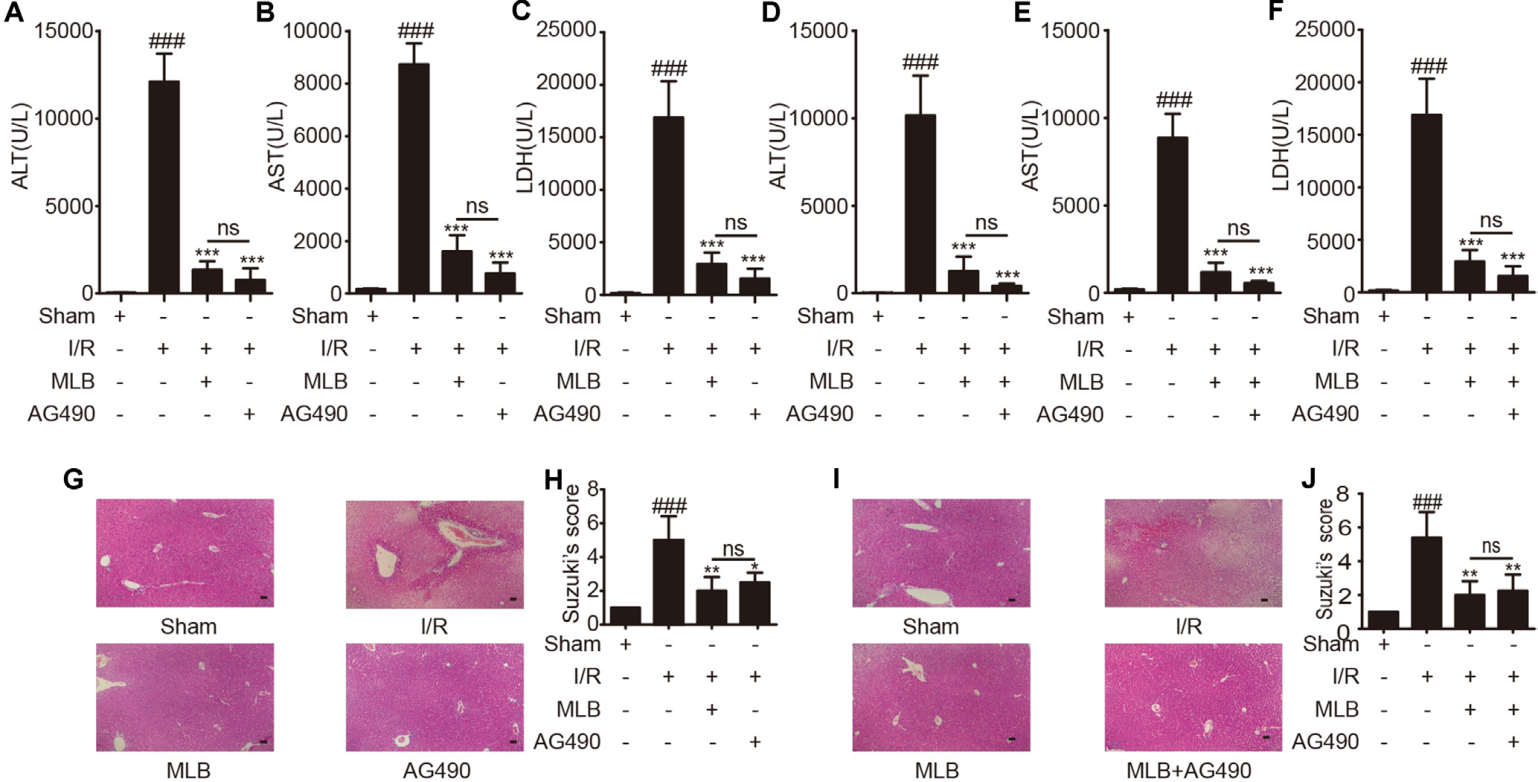
AG490 or MLB+AG490 pretreatment attenuates liver injury caused by ischemia–reperfusion. **(A**–**F)** The levels of ALT, AST, and LDH in sham, I/R, MLB, and AG490 or MLB+AG490 groups. **(G**–**J)** Classical images of sham, I/R, MLB, and AG490 or MLB+AG490 pretreatment groups were examined by H&E staining, and histopathological score was calculated by Suzuki’s scoring standard, magnification x200, scale bar 50μm. Data shown are mean ± SD; n = 3–5 for each group; ns: no statistical significance; ^###^P < 0.001 versus sham group; *P < 0.05, **P < 0.01, and ***P < 0.001 versus I/R group.

In addition, to further explore whether the mechanism of MLB alleviation of hepatic I/R is related to the inhibition of Jak2 signaling pathway, we selected AG490, a specific inhibitor of Jak2, as a positive control or in combination with MLB. The contents of ALT, AST, and LDH (**Figure 5A–C**) were significantly reduced by AG490 pretreatment after I/R surgery, but there was no significant difference compared to the MLB pretreatment group. Thus, we combined MLB with AG490 to further investigate the protective mechanism of MLB. AG490 was given i.p. 1 h prior to MLB treatment. As shown in **Figure 5D–F**, serum ALT, AST and LDH levels were also reduced by MLB+AG490, but there was also no significant difference between the MLB+AG490 group and the MLB group.

Subsequently, we found that liver pathological changes were also significantly reversed in the AG490 pretreatment group compared to the I/R group, and the serious liver pathological changes were also reversed by MLB+AG490, but all the pathological scores in the AG490 or MLB+AG490 group were not significantly different compared to the group with MLB pretreatment alone (**Figure 5G–J**).

### Effect of AG490 or MLB on Oxidative Stress in Liver and Proinflammatory Mediator in Serum

**Figure 6:**
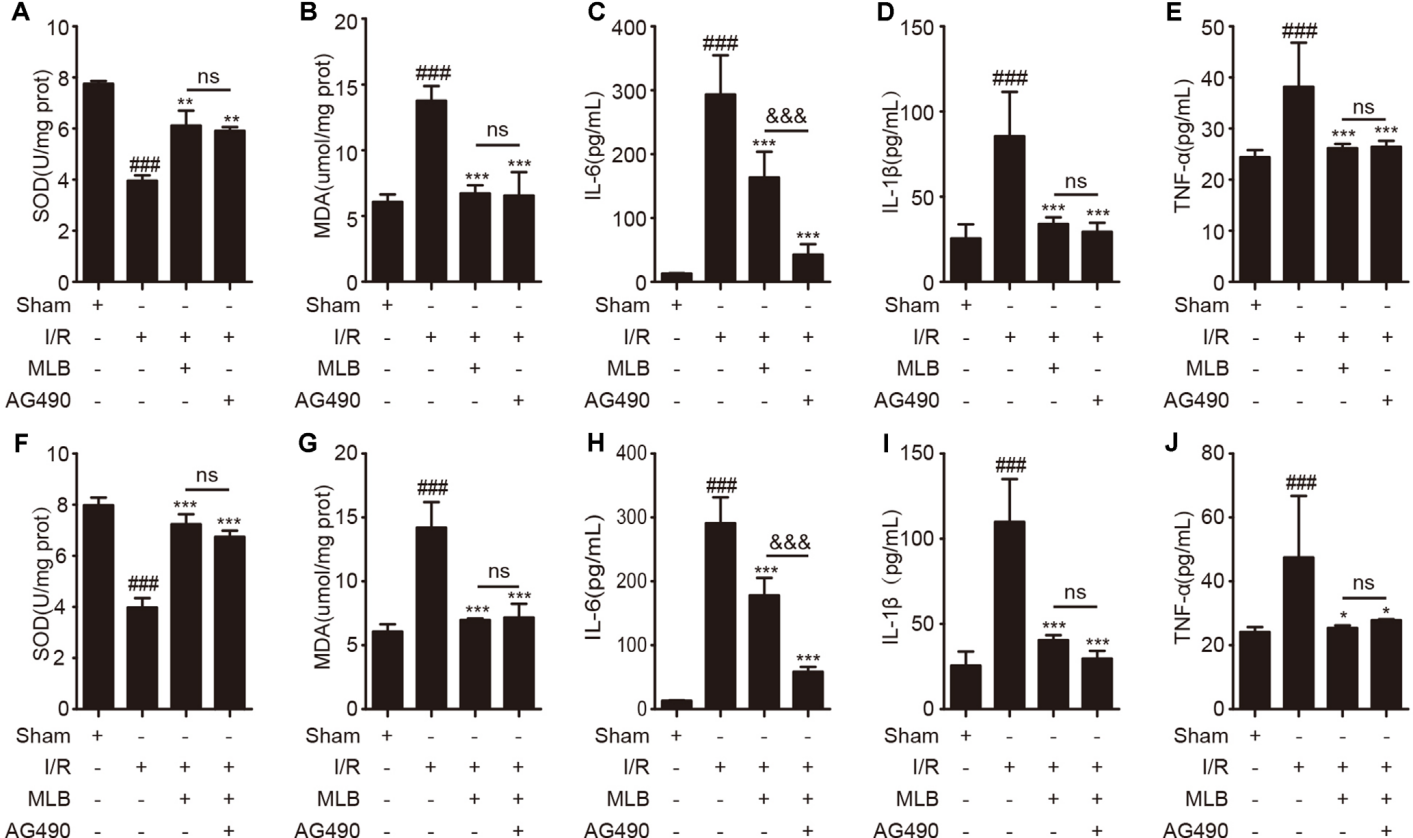
AG490 or MLB+AG490 pretreatment affected oxidative stress in liver and inhibited inflammatory factors’ expression. **(A**, **B)** The expression of SOD and MDA in liver tissue of sham, I/R, MLB, and AG490 groups. **(C**–**E)** Serum IL-6, IL-1β, and TNF-α were detected by ELISA in sham, I/R, MLB, and AG490 groups. **(F**–**G)** The expression of SOD and MDA in liver tissue of sham, I/R, MLB, and MLB+AG490 groups. **(H**–**J)** Serum IL-6, IL-1β, and TNF-α were detected by ELISA in sham, I/R, MLB, and MLB+AG490 groups. The data are expressed as mean ± SD; n = 5 for each group; ns: no statistical significance; ^###^P < 0.001 versus sham group; *P < 0.05, **P < 0.01, and ***P < 0.001 versus I/R group; ^&&&^P < 0.001 versus MLB group.

Next, we repeated assays to detect oxidative stress–related enzymes and proinflammation factors. As shown in **Figure 6A and B**, oxidative stress–related enzymes such as SOD and MDA were altered by Jak2 inhibitor AG490 pretreatment versus those of the I/R group (**Figure 6A and B**). However, AG490 pretreatment did not significantly improve the expression of SOD and MDA in liver tissues compared with the MLB group. Analogously, proinflammatory cytokines including IL-6, IL-1β, and TNF-α were observably decreased by AG490 in serum, as shown in **Figure 6C–E**.

Subsequently, we also investigated the oxidative-stress markers, including SOD and MDA, in the MLB+AG490 pretreatment group compared to the MLB pretreatment group. As shown in **Figure 6F, G**, the expression of SOD and MDA were also altered by MLB+AG490 pretreatment versus the I/R group, while their expression also had no significant difference between the two groups. The levels of proinflammatory mediators were also significantly decreased by MLB+AG490 pretreatment versus MLB pretreatment (**Figure 6H–J**). In this study, we also found that the levels of IL-1β and TNF-α had no significantly difference in the MLB group compared with the AG490 group or MLB+AG490 group, expect for the expression of IL-6 in serum.

### Effect of MLB+AG490 on Jak2/Stat3 Signaling Pathway

**Figure 7:**
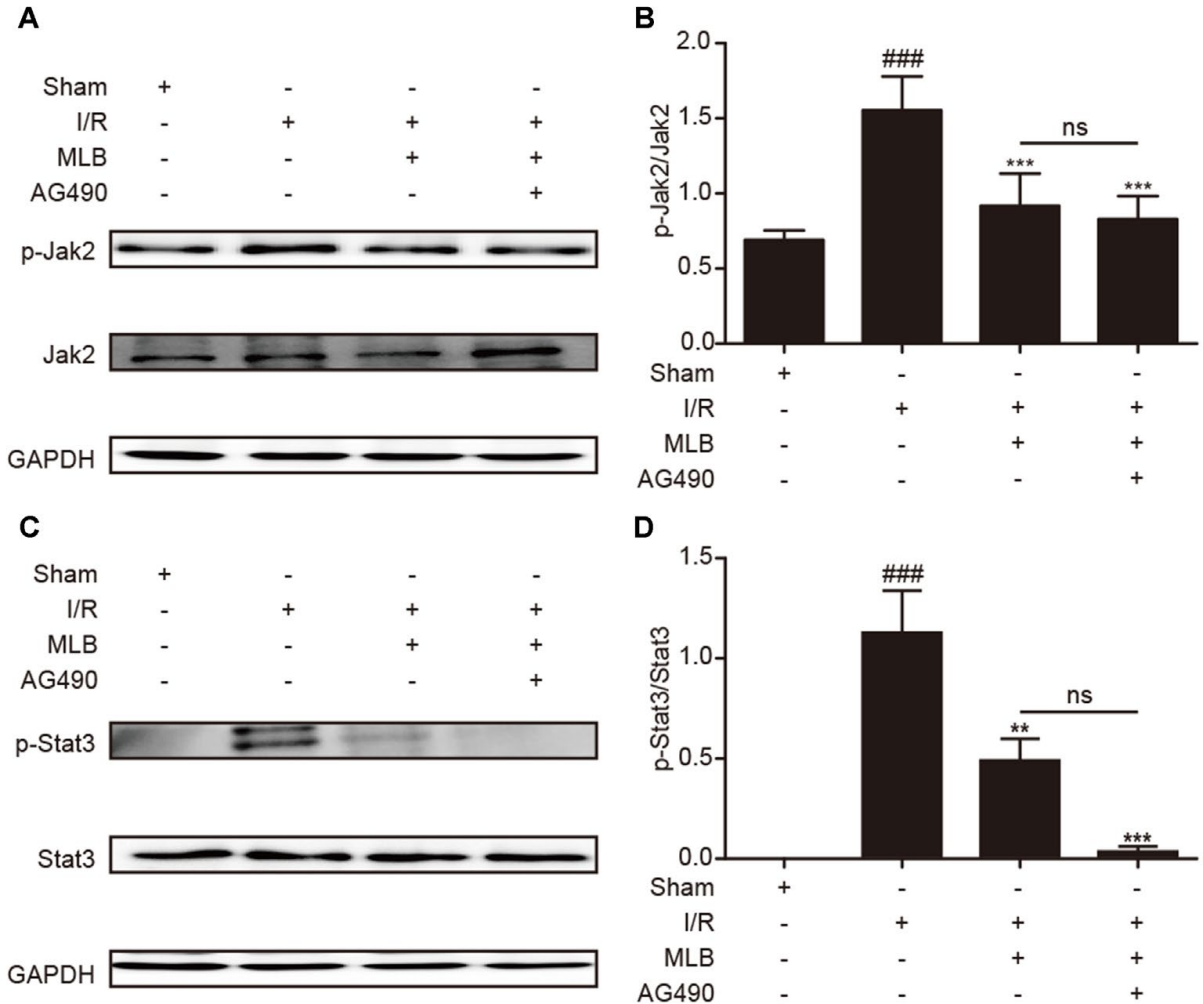
MLB+AG490 pretreatment inhibited Jak2/Stat3 signal pathway in the liver tissue of I/R. **(A)** Western blot was utilized to detect the level of p-Jak2. **(B)** The quantification of p-Jak2/Jak2 was statistically analyzed. **(C)** Western blot was utilized to detect the level of p-Stat3; **(D)** The quantification of p-Stat3/Stat3 was statistically analyzed. The data are expressed as mean ± SD; n = 5 for each group; ns: no statistical significance; ^###^P < 0.001 versus sham group; **P < 0.01, and ***P < 0.001 versus I/R group.

Moreover, to confirm the effect of MLB on Jak2/Stat3, we investigated the levels of proteins such as p-Jak2/Jak2 and p-Stat3/Stat3. As shown in **Figure 7A–D**, the expression levels of p-Jak2/Jak2 and p-Stat3/Stat3 were significantly decreased in the MLB+AG490 group versus the I/R group. However, the levels of these proteins were not significantly different between the MLB group and the MLB+AG490 group. These results suggested that MLB could inhibit the Jak2/Stat3 signaling pathway in the liver, which prevented the liver from the injury by I/R.

### Effect of MLB on Macrophage RAW264.7 Cells

**Figure 8:**
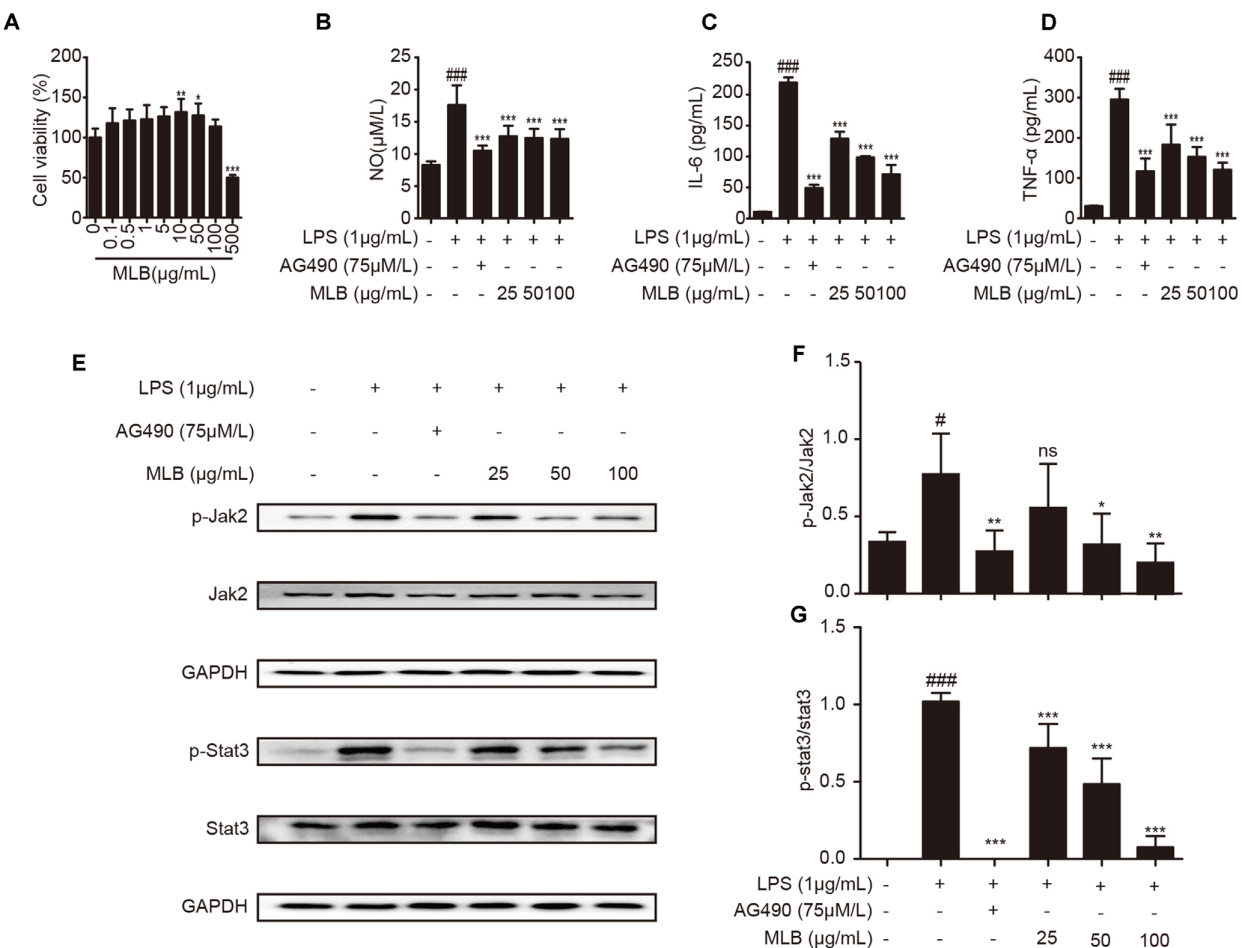
MLB inhibited inflammation *via* Jak2/Stat3 in RAW264.7. **(A)** Cell viability was examined using CCK-8. **(B)** The NO production was measured by Griess assays in cell media, and the levels of IL-6 **(C)** and TNF-α **(D)** were ascertained by ELISA. **(E**–**G)** The levels of the Jak2/Stat3 signaling pathway were measured by Western blotting. All samples were from 5 independent experiments. Each value is expressed as mean ± SD; n = 5 for each group; ns: no statistical significance; ^#^P < 0.05, and ^###^P < 0.001 versus NC group; *P < 0.05, **P < 0.01, and ***P < 0.001 versus LPS group.

To further clarify the anti-inflammatory mechanism of MLB, we verified it in macrophage RAW264.7 cells. First, the cytotoxicity of MLB on RAW264.7 was investigated using a CCK-8 kit. As shown in **Figure 8A**, there was no cytotoxicity at concentrations of 0–100 *μ*g/ml of MLB. The cell viability was increased at concentrations of 25–50 *μ*g/ml of MLB. As shown in **Figure 8B**, the NO content was significantly increased after LPS stimulation compared to that of the native control (NC) group.

Proinflammatory factors such as IL-6 and TNF-α were also investigated by ELISAs in cell media. The levels of IL-6 (**Figure 8C**) and TNF-α (**Figure 8D**) were significantly elevated after 8-h treatment with LPS compared to NC. MLB dose-dependently reduced the concentrations of IL-6 and TNF-α.

To further analyze the effect of MLB on Jak2/Stat3, we performed Western blotting to measure the levels of characteristic proteins in the Jak2/Stat3 pathway, including p-Jak2, Jak2, p-Stat3, and Stat3. As shown in **Figure 8E–G**, LPS stimulation observably may increase the ratio of p-Jak2/Jak2 and p-Stat3/Stat3 in RAW264.7 cells. However, MLB pretreatment strikingly inhibited the expressions of p-Jak2/Jak2 and p-Stat3/Stat3 due to LPS-induced elevation. These results also suggest that the anti-inflammatory effect of MLB is closely related to the inhibition of the Jak2/Stat3 signaling pathway.

## Discussion

MLB is a hydrophilic constituent of *Salvia miltiorrhiza*, which was widely used for the treatment of cardiovascular and cerebrovascular disease (Zhu Jinqiang, 2010). As the natural existing form in the herb, MLB is different from its free acid form, salvianolic acid B (or lithospermic acid B) in its physicochemical properties and its bioacticity because the chelating magnesium stabilizes the molecule and makes its conformation different. MLB possesses multitudinous pharmacological properties, including antioxidative, anti-inflammatory, and antiapoptotic effects, in various experimental disease models (Wu and Wang, 2012). MLB was shown to inhibit inflammatory mediator production *via* regulation of nuclear factor-kappa B signaling pathways in activated T cells (Cheng et al., 2012). Moreover, MLB was shown to protect against stroke by upregulating p-Akt (Cao et al., 2015). The reports about MLB in liver diseases are limited. Paik et al. (2011) found that MLB had an antifibrotic effect in thioacetamide (TAA)-induced cirrhotic rats *via* inhibiting fibrogenic responses. However, the protective effect of MLB on hepatic I/R needs more research. In this study, we found that MLB can protect the liver from HIRI by inhibiting the Jak2/Stat3 signaling pathway. To our knowledge, this is the first report demonstrating that MLB attenuates liver I/R injury through Jak2 inhibition.

HIRI is a common pathological phenomenon and an acute injury with high morbidity and mortality during liver surgery, liver transplantation, and hepatectomy (Arkadopoulos et al., 2011; Douzinas et al., 2012; Lu et al., 2018). Liver function was impacted after I/R surgery (Nakazato et al., 2018). The drug could not get into the ischemic liver to reduce injury during the ischemia period. Therefore, the present study assessed the impact of MLB pretreatment on hepatic function and its therapeutic efficacy against liver I/R injury. The data showed that hepatic I/R aggravated the contents of ALT, AST, and LDH and increased histopathological features compared to the sham group, indicating liver dysfunction and hepatocyte damage induced by I/R in mice. We also found that MLB had no adverse effect on liver function (ALT, AST, and LDH) compared with the sham treatment. MLB pretreatment significantly protected the mice against liver I/R injury, as confirmed by alterations in abnormal liver function and histopathology.

In the process of ischemia, some parenchymal hepatic cells are killed because of metabolic dysfunction due to hypoxia and innutrition (Zhai et al., 2013). Oxidative stress plays an important role during I/R (Ma and Jin, 2019; Yao et al., 2019). SOD activity is closely related to oxygen free radical clearance and lipid oxidation resistance, and all of these indicators are also important markers of I/R injury (Okado-Matsumoto and Fridovich, 2001). If the SOD activity is inhibited during I/R, MDA will be produced due to lipid oxidation in cells, and the balance of antioxidants and oxidants will also be disturbed (Demiryilmaz et al., 2014; Wang et al., 2015b). The present experiment showed that MLB pretreatment could increase SOD activity and decrease MDA production in the process of hepatic I/R.

When the blood supply is restored, there is a shift from metabolic dysfunction caused by ischemia to an excessive innate immune response caused by reperfusion. After DAMP recognition by the TLRs on the membrane of the KCs, excessive proinflammatory mediators are secreted during the I/R injury (Langdale et al., 2008; Harari and Liao, 2010; Zhao et al., 2018). Our data showed that the proinflammatory cytokines, including IL-6, IL-1β, and TNF-α, were abnormally elevated in the serum during liver I/R. Our results are consistent with numerous research reports showing that increased contents of inflammatory factors were induced by the I/R injury (Walsh et al., 2009; Van Golen et al., 2012; Gendy et al., 2017; Rong et al., 2017). A clinical study found that KC activation was inhibited using glycine in human liver transplantation and that the damage caused by I/R was also reduced (Schemmer et al., 2001). Meanwhile, John D. et al. (Lang et al., 2014) found that preemptive inhaled nitric oxide could protect against I/R injury and reduce the inflammatory effects during human liver transplantation. Therefore, controlling the inflammatory response may be helpful for the prevention and treatment of HIRI. Our data indicated that the production of inflammatory mediators was significantly reduced by MLB pretreatment after I/R surgery.

According to a previous report, the Jak2/Stat3 pathway is an important signaling pathway that has been confirmed to regulate the inflammatory response during I/R injury (Si et al., 2014; Luo et al., 2016). The Jak/Stat signaling pathway is activated by proinflammatory cytokines when excessive proinflammatory cytokines such as interleukin and interferon are secreted (Aaronson and Horvath, 2002; Banerjee et al., 2017; Li et al., 2018). The expression of inflammatory cytokines could also be regulated by the Jak2/Stat3 signal pathway (Zhou et al., 2016; Kim et al., 2017; Zhang et al., 2017; Li et al., 2018). Therefore, the efficacy of MLB on Jak2/Stat3 signaling was investigated in a mouse hepatic I/R model. Jak2 was activated *via* phosphorylation during I/R injury (Freitas et al., 2010; Luo et al., 2016; Zhao et al., 2016; Hu et al., 2017). The data showed that I/R significantly increased the level of p-Jak2 compared to that of the sham group. We found that Jak2 activation was significantly suppressed by pretreatment of mice with MLB, and the level of p-Jak2 was significantly reduced in the MLB pretreatment group compared with the I/R group. Stat3 is the most widely studied member of the Stat family of proteins, which is closely related to the Jak family of proteins, and cytosolic Stat3 undergoes phosphorylation following Jak2 activation (Banerjee et al., 2017; Bousoik and Montazeri Aliabadi, 2018). Xiong et al. (2018) also found that when the Stat3 activation was depressed in hepatic I/R injury, hepatic injury was alleviated. Han et al. (2018) reported that Stat3 upregulation or activation is one critical molecular mechanism of hepatic I/R injury. The level of p-Stat3 was detected in the ischemic liver, and p-Stat3 level was abnormally elevated in the I/R group versus the sham group. And p-Stat3 was significantly lower in mice pretreated with MLB than mice in the I/R group.

To further clarify the anti-inflammatory mechanism of MLB, we selected Jak2 inhibitor AG490 as a positive control. Mascareno et al. (2001) found that interfering with activation of the Jak/Stat pathway promotes recovery in cardiac function. Freitas et al. (2010) also showed that the blockade of the Jak2 signaling pathway ameliorates mouse liver injury induced by I/R. In this study, we found that the liver I/R injury was reduced by blockade of Jak2 activation using AG490, while all results of the AG490 pretreatment group showed no significant difference from those of the MLB pretreatment group, expect for IL-6. To confirm the correlation between the anti-inflammatory effect of MLB and Jak2, mice were pretreated using MLB+AG490. Compared with the I/R group, MLB+AG490 pretreatment could recover abnormal liver function and reduce the degree of liver tissue damage, inflammatory response, and oxidative stress caused by I/R, but there was no significant difference between the MLB group and the I/R group. Moreover, MLB+AG490 significantly inhibited the Jak2 and Stat3 activation. These results suggest that the anti-inflammatory mechanism of MLB was related to the inhibition of Jak2.

RAW264.7 cells, as murine macrophages, were selected for this study. According to a previous report, macrophages or immune systems are activated by some toxicant, such as LPS (Hsu and Wen, 2002; Lawrence et al., 2002; Pestka and Zhou, 2006). Therefore, we established an *in vitro* model in RAW264.7 cells with LPS stimulation. After the LPS stimulation, the inflammatory mediators, including NO, IL-6, IL-1β, and TNF-α, were secreted from the macrophage (Yun et al., 2008; Ham et al., 2015). MLB pretreatment could reverse these abnormal phenomena. Research also finds that Jak2 and Stat3 are specifically activated by IL-6 or INF-γ (Ivashkiv and Hu, 2003; Murray, 2007). We also observed that IFN-γ reversed the MLB's inhibition effect on content of NO and IL-6 after LPS stimulation (**Figure S2A** and **B**). LPS provoked various signal pathways including Jak/Stat (Okugawa et al., 2003; Li et al., 2019). In this study, we also observed that the levels of p-Jak2 and p-Stat3 were abnormally elevated after the LPS stimulation. MLB pretreatment significantly decreased these effects in a dose-dependent manner. The *in vivo* results were confirmed by these *in vitro* findings.

**Figure 9:**
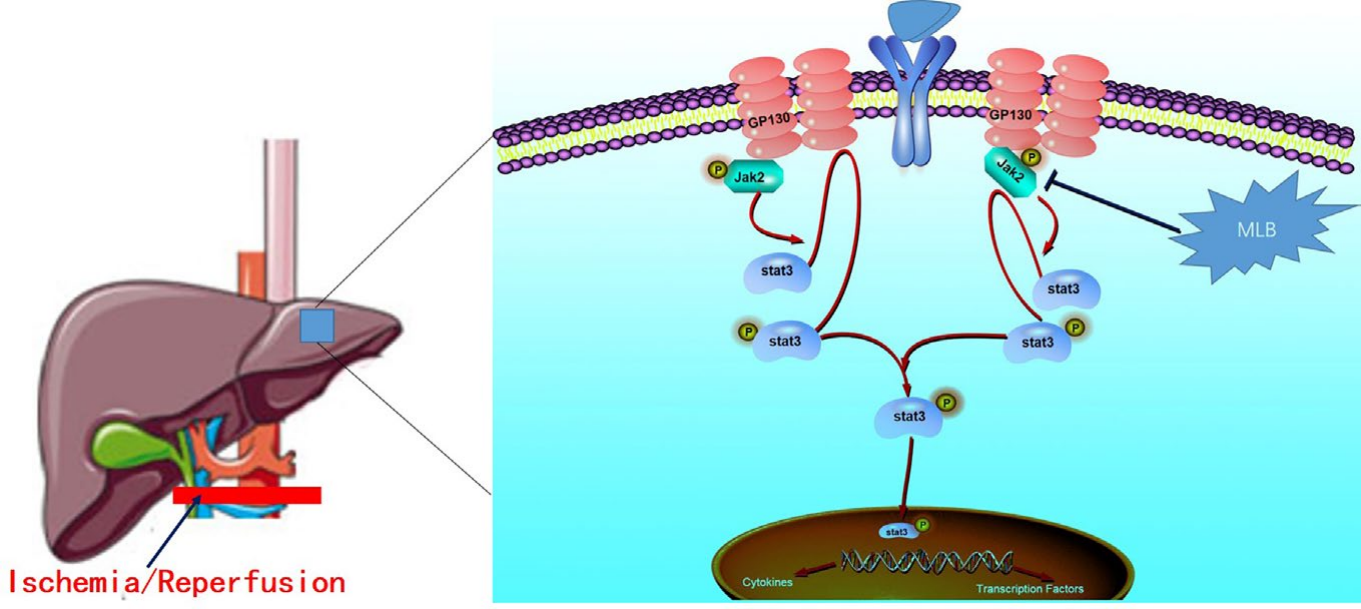
The protective mechanism of MLB against hepatic I/R. The Jak2/Stat3 signaling pathway was activated when excessive proinflammation mediators were secreted, and then the inflammatory cytokines were further expressed. MLB could inhibit the activation of the Jak2/Stat3 signaling pathway and decrease the pro-inflammatory cytokines to reduce the hepatic I/R injury.

In conclusion, we identified the protective effect of MLB in hepatic I/R injury and a key mechanism underlying the hepatoprotective properties of MLB (**Figure 9**). Mechanistic studies suggested that MLB pretreatment could inhibit Jak2/Stat3 signaling pathway activation, which contributes to its liver-protective role in liver I/R injury. Collectively, these data support the conclusion that MLB pretreatment may be used as an alternative therapy for the prevention of liver I/R injury in clinical practice.

## Data Availability Statement

All datasets generated for this study are included in the manuscript and/or the supplementary files.

## Ethics Statement

This study was carried out in accordance with the recommendations of Institutional Animal Care and Use Committee (IACUC), Shanghai Institute of Materia Medica (SIMM). The protocol was approved by the Institutional Animal Care and Use Committee (IACUC), Shanghai Institute of Materia Medica (SIMM).

## Author Contributions

NZ, LH, YX, GP, and QF conceived and designed the experiments. QD, ZW, HP, YZ, and LX assisted with the experiments. NZ wrote the paper. LH, YX, ZW, HP, LX, YZ, and GP critically revised the manuscript. All the authors read and reviewed the final manuscript.

## Funding

This study was supported by the Independent Deployment Program of the Institute of Pharmaceutical Innovation of the Chinese Academy of Sciences (grant CASIMM0120184005), the National Science Foundation of China (grant 81872927), and the “Organ Reconstruction and Manufacturing” Strategic Priority Research Program of the Chinese Academy of Sciences (grant XDA16020205).

## Conflict of Interest Statement

The authors declare that the research was conducted in the absence of any commercial or financial relationships that could be construed as a potential conflict of interest.
