# Supplementary material for: The Protective Effect of Magnesium Lithospermate B on Hepatic Ischemia/Reperfusion via Inhibiting the Jak2/Stat3 Signaling Pathway
Source: Front Pharmacol. 2019 May 31;10:620. doi: 10.3389/fphar.2019.00620 (PMC6558428; doi:10.3389/fphar.2019.00620)
Supplement: Supplementary file 1 [file DataSheet_1.pdf]

frontiers

Supplementary Material

1. Supplementary Figures

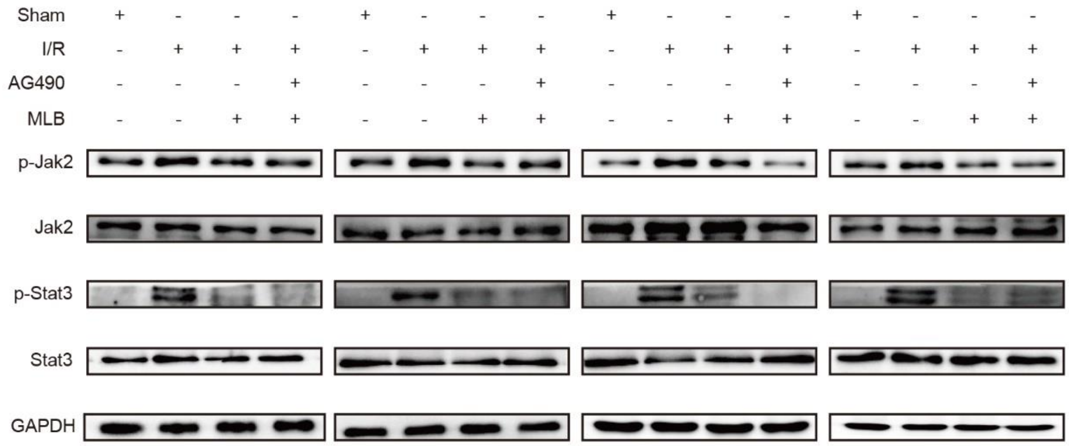

Supplementary Figure 1. MLB+AG490 pretreatment inhibited Jak2/Stat3 signal pathway in the liver tissue of I/R (This study was repeated five times. One figure was listed in figure 7. The rest four independent studies were listed as sFigure 1).

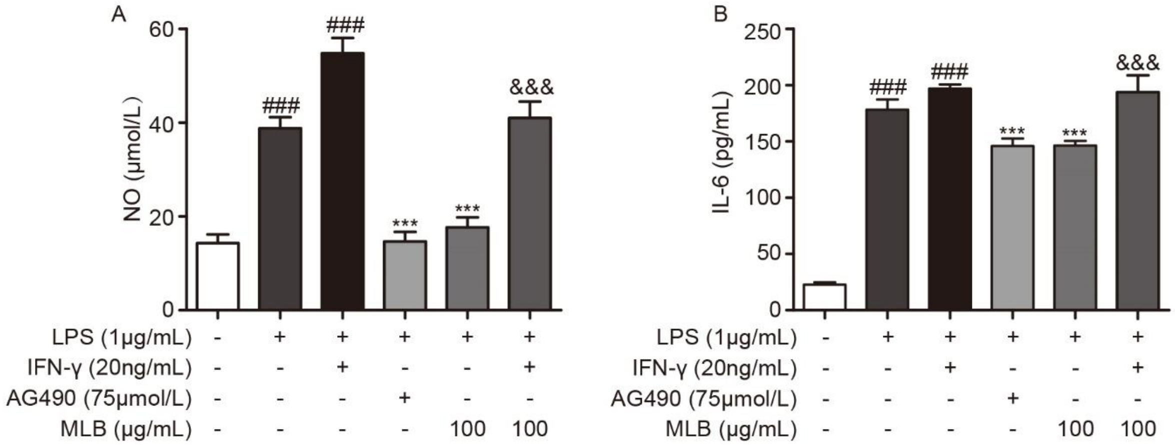

Supplementary Figure 2. IFN-γ reversed the inhibition of MLB in RAW264.7 cells. (A) The content of NO in different groups. (B) The secretion of IL-6 in different groups. Each value is expressed as mean ±S.D, n=4 for each group. $^{###}$P<0.001 versus NC group, ***P<0.001 versus LPS group, $^{\&\&\&}$P<0.001 versus MLB group.

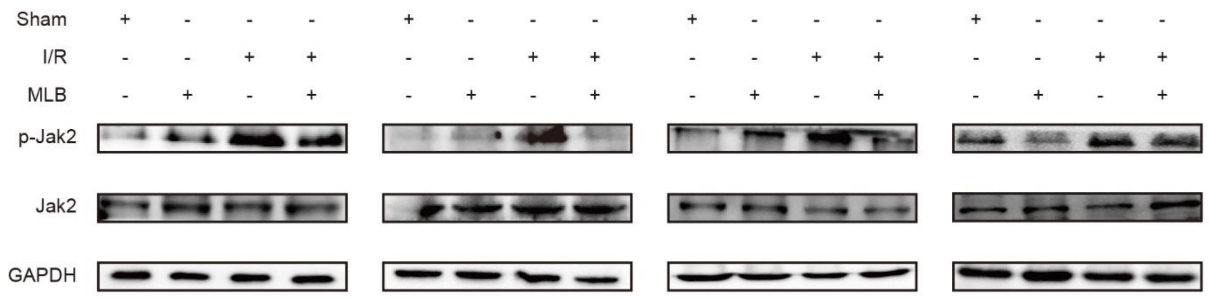

Supplementary Figure 3. MLB pretreatment inhibited Jak2/Stat3 signal pathway in the liver tissue of I/R. (This study was repeated five times. One figure was listed in figure 4A. The rest four independent studies were listed as sFigure 3).
